# Therapeutic Insights in Chronic Kidney Disease Progression

**DOI:** 10.3389/fmed.2021.645187

**Published:** 2021-02-23

**Authors:** Amir Shabaka, Clara Cases-Corona, Gema Fernandez-Juarez

**Affiliations:** Nephrology Department, Hospital Universitario Fundación Alcorcón, Madrid, Spain

**Keywords:** chronic kidney disease, end-stage kidney disease, drug therapy, SGLT2 inhibitors, renal fibrosis, inflammation

## Abstract

Chronic kidney disease (CKD) has been recognized as a leading public health problem worldwide. Through its effect on cardiovascular risk and end-stage kidney disease, CKD directly affects the global burden of morbidity and mortality. Classical optimal management of CKD includes blood pressure control, treatment of albuminuria with angiotensin-converting enzyme inhibitors or angiotensin II receptor blockers, avoidance of potential nephrotoxins and obesity, drug dosing adjustments, and cardiovascular risk reduction. Diabetes might account for more than half of CKD burden, and obesity is the most important prompted factor for this disease. New antihyperglycemic drugs, such as sodium-glucose-cotransporter 2 inhibitors have shown to slow the decline of GFR, bringing additional benefit in weight reduction, cardiovascular, and other kidney outcomes. On the other hand, a new generation of non-steroidal mineralocorticoid receptor antagonist has recently been developed to obtain a selective receptor inhibition reducing side effects like hyperkalemia and thereby making the drugs suitable for administration to CKD patients. Moreover, two new potassium-lowering therapies have shown to improve tolerance, allowing for higher dosage of renin-angiotensin system inhibitors and therefore enhancing their nephroprotective effect. Regardless of its cause, CKD is characterized by reduced renal regeneration capacity, microvascular damage, oxidative stress and inflammation, resulting in fibrosis and progressive, and irreversible nephron loss. Therefore, a holistic approach should be taken targeting the diverse processes and biological contexts that are associated with CKD progression. To date, therapeutic interventions when tubulointerstitial fibrosis is already established have proved to be insufficient, thus research effort should focus on unraveling early disease mechanisms. An array of novel therapeutic approaches targeting epigenetic regulators are now undergoing phase II or phase III trials and might provide a simultaneous regulatory activity that coordinately regulate different aspects of CKD progression.

## Introduction

Chronic kidney disease (CKD) has been recognized as a leading public health problem worldwide. The global estimated prevalence of CKD is 13.4% (11.7–15.1%) (1) CKD has a powerful impact on global morbidity and mortality by increasing the risk of cardiovascular diseases, diabetes, and hypertension. The Global Burden of Diseases study estimated that 1.2 million deaths were due to kidney failure, and 19 million disability-adjusted life-years (DALYs) as well as 18 million years of life lost from cardiovascular diseases were directly attributable to CKD (2). The DALYs associated with CKD have increased significantly in the last three decades (3). Even in early stages, CKD has been associated with an increased cardiovascular morbidity and mortality in both the general population as well as those patients with increased risk of CVD, therefore early detection of CKD as well as retarding the progression of kidney disease is deemed essential to reduce cardiovascular morbimortality as well as the economic burden caused by kidney disease (4).

**Figure 1:**
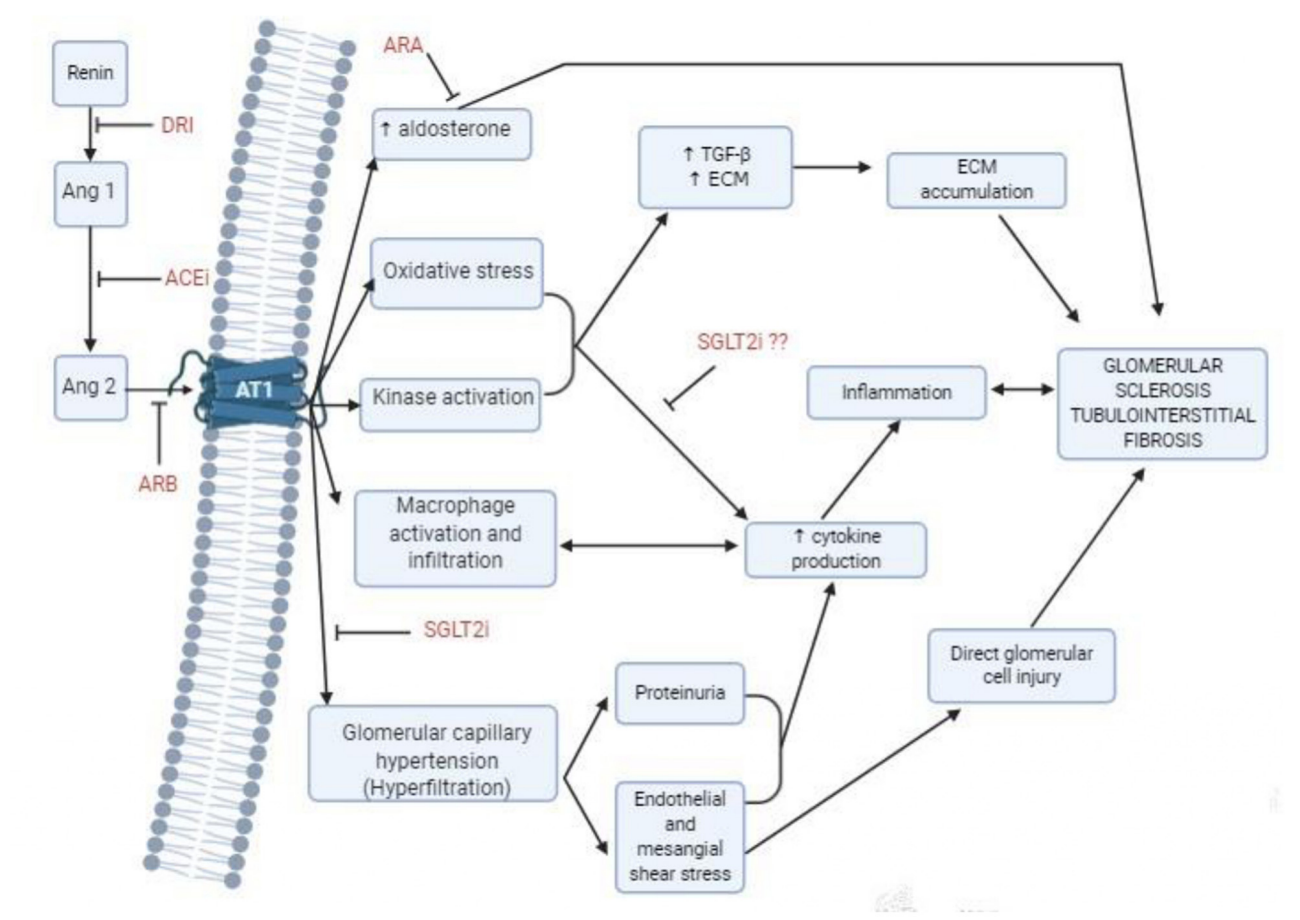
Mechanism of progression of chronic kidney disease, and treatment options to delay progression. ACEi, Angiotensin converting enzyme inhibitors; Ang, Angiotensin; ARA, Aldosterone receptor antagonists; ARB, Angiotensin receptor blockers; AT1, Angiotensin II Type 1 receptor; DRI, Direct renin inhibitors; SGLT2i, Sodium-glucose cotransporter 2 inhibitors.

It has been well-acknowledged that when the glomerular filtration rate (GFR) decreases below a critical level, CKD continues to progress unabatingly toward end-stage kidney disease (ESKD). The loss of a critical number of nephrons causes a vicious cycle of further nephron loss, and this damage is perpetuated even when the underlying cause of the disease is treated (5). There are several interconnected mechanisms that are involved in the progression of CKD, including hemodynamic and non-hemodynamic changes. The first occur in glomeruli, involving an increased glomerular capillary hydrostatic pressure and increased single-nephron glomerular filtration load, inducing glomerular injury and indirectly tubular injury. Hyperfiltration induces direct endothelial cell damage, increasing wall stress that may cause detachment and podocyte loss, and increased strain on the mesangial cells that can stimulate them to produce cytokines and extracellular matrix, such as transforming growth factor β (TGF-β) or isoforms of platelet-derived growth factor (6, 7). Figure 1 summarizes the pathophysiological mechanisms of CKD progression. The mechanism by which tubulointerstitial fibrosis develops is incompletely understood. Fibrosis is part of the normal repair process that is triggered in response to injury, however, deregulation of this process leads to pathological accumulation of extracellular matrix proteins, mainly collagens. These processes result in the replacement of parenchymal tissue by extracellular matrix (8). Supplementary Figure 1 shows how the interactions of different risk factors contribute in the progression of CKD.

**Figure 2:**
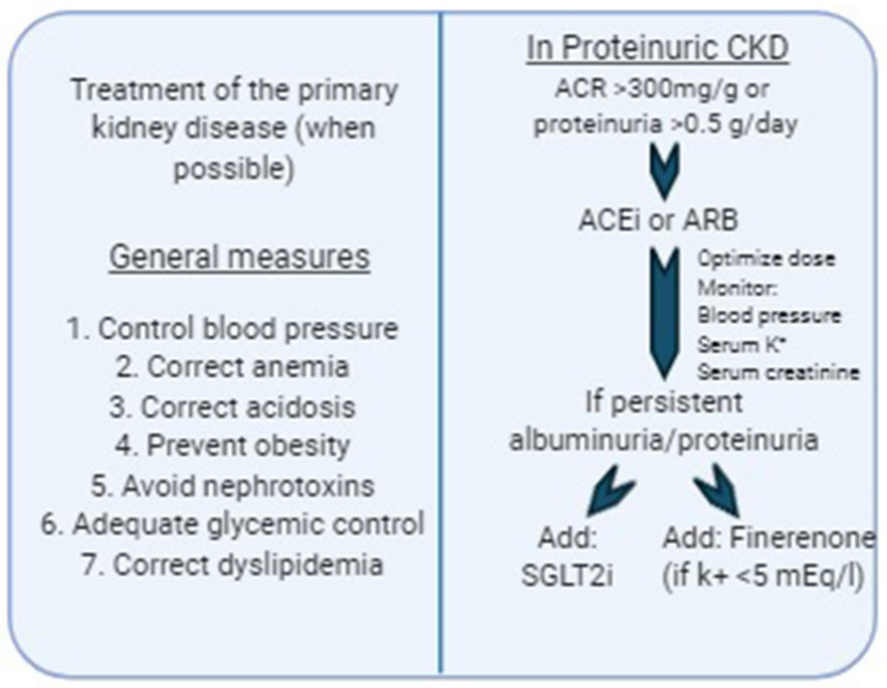
Therapeutic approach for delaying CKD progression.

In the last few decades some therapeutic agents have been identified as useful in retarding the progression of CKD. Several clinical trials have demonstrated that both renin-angiotensin-aldosterone (RAS) system inhibitors and more recently, agents that inhibit sodium-glucose cotransporter 2 (SGLT2) in the proximal convoluted tubule, reduce the loss of GFR in the long-term. The main mechanism of their action is based on the reduction of intraglomerular pressure, therefore proteinuric CKD get the most benefit from these therapies. On the other hand, and despite some positive results obtained from preclinical studies, there are no established strategies to modulate inflammation or delay progression of tubulointerstitial fibrosis. Hence, although several new therapeutic agents have been developed recently that look promising for the prevention of CKD progression, it is certainly necessary to develop agents that target different components of the fibrogenic cascade. Figure 2 shows a proposed algorithm for prevention of CKD progression according to the current available evidence.

In this article we review the therapeutic targets for preventing CKD progression, focusing in the latest developments in treatment approaches for delaying CKD progression, we reflect on potential new biomarkers and novel therapeutic targets, as well as future lines of treatment, including regenerative therapies.

## RAS Blockade

### Angiotensin-Converting Enzyme Inhibitor and Angiotensin Receptor Blocker Monotherapy

RAS blockade with angiotensin converting enzyme inhibitors (ACEi) or angiotensin receptor blockers (ARBs) is the cornerstone therapy to reduce proteinuria, CKD progression, and cardiovascular risk. These benefits appear to be comparable between ACEI and ARBs when they are used in equivalent doses, and carry comparable adverse effects besides cough which is exclusive to ACEi.

RAS blockade has been shown to be renoprotective in diabetic patients with microalbuminuria or overt nephropathy, as well as in non-diabetic CKD. There is an increasing body of evidence which shows that RAS blockade at doses higher than the maximum antihypertensive dose may afford additional renoprotection. In the SMART study, 269 patients who had persistent proteinuria (>1 g/day) despite seven weeks of treatment with dose of candesartan (16 mg/day) were randomized to receive candesartan at a dose of 16, 64, or 128 mg/day. After 30 weeks, the maximal dose of candesartan (128 mg/day) achieved an additional decrease in proteinuria of 33%. Reductions in BP were not different across the three treatment groups, although elevated serum potassium levels (K^+^ >5.5 mEq/L) led to the early withdrawal of 11 patients (9).

### Combination Angiotensin-Converting Enzyme Inhibitor and Angiotensin Receptor Blocker Therapy

Theoretically, the inhibition of RAS system at several different steps of the pathway would lead to a more complete inhibition and therefore was thought to maximize the renoprotective effects of RAS blockade. In several small clinical studies, more complete inhibition of the RAS with combination ACEI and ARB treatment was associated with additional lowering of proteinuria. A large trial of combination therapy in patients with hypertension and increased cardiovascular risk reported no additional benefit with respect to cardiovascular outcomes, and although combination therapy was associated with a greater reduction in proteinuria than monotherapy, they also induced a higher incidence of acute renal failure. It should be noted, however, that subjects were selected on the basis of cardiovascular risk profile and that the majority did not have reduced GFR or proteinuria. A similar study in patients with type 2 diabetes and albuminuria over 300 mg/g also found no benefit with respect to the primary outcome of CKD progression, ESKD, or death in participants randomized to combination ACEi and ARB therapy vs. monotherapy. Again, those patients receiving combination therapy showed a significantly higher incidence of acute kidney injury (AKI) and hyperkalemia. However, in neither of the two studies was dual therapy compared to an equipotential dose of either drug in monotherapy, and therefore the dual blockade effect could not be analyzed separately from the effect carried by the use of different dosage. In the PRONEDI trial, in patients with overt diabetic nephropathy, the combination therapy consisting of lisinopril 20 mg plus irbesartan 300 mg was compared with the use of lisinopril at a dose of 40 mg and irbesartan at a dose of 600 mg in monotherapy. In this study, proteinuria decreased in the three groups similarly, with no differences in CKD progression or adverse effects (including acute kidney injury and hyperkalemia) after a median follow-up of 32 months, indicating that optimization of RAS blockade depends on dosage rather than the use of a single or combined RAS blocker (10).

Our goal should be the maximum well-tolerated RAS blockade dose, regardless of whether it is achieved with ARB, ACEI in monotherapy or their combination.

### Renin Inhibitor Therapy

The development of direct renin inhibitors made it possible to inhibit the RAS at its rate-limiting step (the conversion of angiotensinogen to angiotensin I) and thereby to achieve more complete blockade. Two early randomized trials reported additional lowering of albuminuria in subjects with diabetic nephropathy receiving the combination of a direct renin inhibitor and ARB therapy vs. ARB therapy alone (11, 12). However, a large randomized trial (ALTITUDE) that included 8,561 people with type 2 diabetes and albuminuria or cardiovascular disease was stopped prematurely. Despite greater lowering of blood pressure and albuminuria with the combination of direct renin inhibitors and ARB therapy, no benefit was observed with respect to cardiovascular events, CKD progression, ESKD, or death vs. ARB monotherapy. Additionally, combination therapy was associated with a higher incidence of hyperkalemia and hypotension (13).

Therefore, Aliskiren may be a suitable alternative RAS blocker for those intolerant to either ACE inhibitors or ARBs.

### Use of RAS Blockade in the Geriatric Population and in Advanced CKD

It is not known whether the benefits from RAS inhibition in proteinuric CKD can be extended to elderly patients, since they are often underrepresented in clinical trials and their risk for experiencing the outcome of interest during their remaining lifetime may be different than for the younger population. In a study that included 790,342 patients over 70 years old, the number of patients necessary to treat to prevent one case of ESKD ranged from 2,500 among patients with an GFR 60 ml/min/1.73 m^2^ and negative or trace proteinuria and GFR 45–59 ml/min/1.73 m^2^ and no dipstick proteinuria to 16 among those with GFR 15–29 ml/min/1.73 m^2^ and ≥2+ dipstick proteinuria. Overall, 91% of patients included in this large series should be considered as having a “low or moderate risk for kidney disease progression” and the number of patients necessary to treat to avoid an event was >100 (14).

These findings remind us that the benefit obtained from RAS inhibition for renoprotection occurs in patients with protein excretion of >1g/day, while the vast majority of patients over the age of 70 years with CKD do not belong to this category. In fact, they would be unnecessarily exposed to the adverse effects of RAS inhibition.

Another important question is whether the benefit from ACEi or ARBs extends to patients with advanced proteinuric CKD, in which the risk of hyperkalemia is significantly increased. This point was addressed in a study in which 422 patients with non-diabetic CKD were assigned to benazepril or placebo on top of standard of care. A specific group of patients (*n* = 281) included in this study had advanced CKD with serum creatinine between 3.1 and 5 mg/dL. The benefit from ACE inhibitors in terms of reaching ESKD or doubling of serum creatinine was present even in those patients with advanced CKD and especially when proteinuria was above 1 g/day. Serious hyperkalemia was similar with benazepril and placebo (15). However, the results of this study are not generalizable to all patients with proteinuric CKD, since the patients were rigorously selected and closely monitored during the study for potassium control, and also dietary intake of potassium was likely lower than that in Western patients.

### Non-hemodynamic Effects of Angiotensin II

Angiotensin II (Ang II) appears to participate in the development of tubulointerstitial fibrosis, mediated through one of the Ang II type 1 receptors that are present in the glomerulus. Ang II also contributes in cytokine and chemokine-mediated recruitment of inflammatory cells into the kidney. Overall, these effects can generate profibrotic factors such as TGF-β, connective tissue growth factor, epidermal growth factor (EGF), and other chemokines. In fact, regression of glomerulosclerosis has been observed in rodents on RAS blockade but this phenomenon has not been observed in humans.

Clinical trials have not specifically addressed the question of whether RAS blockade decreases renal fibrosis. In some *post-hoc* studies, some biomarkers of inflammation and serum fibrosis, such as interleukin 6 (IL-6) or Dickkopf 3 (DKK3), were measured sequentially; treatment with RAS blockade did not modify their levels (Sanchez-Alamo et al., submitted). Kidney tissue studies are likely needed to describe the local mechanisms that pass unnoticed in serologic test studies (Table 1).

**Table 1:** Summary of the main clinical trials studying RAS blockade, aldosterone antagonism, endothelin antagonism, and bicarbonate therapy in delaying CKD progression.

**Clinical Trials**	**Studied agents**	**Year**	***n***	**Baseline GFR (ml/min/1.73 m^**2**^)**	**Baseline proteinuria**	**Patients**	**Follow-up period**	**Primary outcomes**	**Results**
**RAS blockade**									
Lewis et al. (16)	Captopril vs. placebo	1993	409	84 ± 46	2,500 ± 2,500 g/day	DM nephropathy with ACR ≥ 500 mg/g and SCr <2.5 mg/dl	3 years	SCr doubling ESKD or death	Risk reduction 48% (16–69%, *p =* 0.006) Risk reduction 50% (18–70%, *p =* 0.007)
IDNT (17)	Irbesartan vs. amlodipine vs. placebo	2001	1,715	SCr 1.7 ± 0.5 mg/dl	2.9 (1.6–5.4)	DM nephropathy	2.6 years	SCr doubling, ESKD or Death	Risk reduction 20% vs. placebo (*p =* 0.02), 23% vs. amlodipine (*p =* 0.006)
RENAAL (18)	Losartan vs. placebo	2001	1,513	SCr 1.9 ± 0.5 mg/dl	ACR 1237 mg/g	DM nephropathy	3.4 years	SCr doubling, ESKD, or Death	Risk reduction 16% (2–28%), *p =* 0.02
ABCD (19)	Enalapril vs. nisoldipine	2000	470	81.8 ± 7.1	6.3 ± 0.2 mg/g	Normotensive Type 2 DM	5.3 years	Change in SCr	NS
ONTARGET (20)	Ramipril + Telmisartan vs. monotherapy	2008	25,620	73.6 ± 19.6	ACR 7.2 (6.9–7.4) mg/g	Type 2 DM with end-organ damage or atherosclerotic vascular disease	56 months	SCr doubling, ESKD, or death	HR = 1.09 [1.01–1.18]
VA-NEPHRON (21)	Lisinopril + Losartan vs. monotherapy	2013	1,448	53.6 ± 15.5	ACR 842 (495–1,698) mg/g	DM nephropathy	2.2 years^*^	GFR decline ≥30 ml/min if baseline >60 ml/min, or >30% if baseline <60 ml/min, ESKD, or death	HR = 0.88 [0.70–1.12]
ALTITUDE (13, 22)	Aliskiren + ACEi or ARB vs. monotherapy	2012	8,561	57 ± 22	ACR 206 (57–866) mg/g	Type 2 DM with CKD or CVD	32.9 months^*^	SCr doubling, ESKD, or death	HR = 1.03 [0.87–1.23]
PRONEDI (10)	Lisinopril + Irbesartan vs. monotherapy	2013	133	49 ± 21	1.32 g/24 h (1.1–1.62)	DM nephropathy	32 months	>50% increase in baseline SCr, ESKD, or death	HR = 0.96 [0.44–2.05] vs. lisinopril HR = 0.90 [0.39–2.02] vs. irbesartan
AASK (23)	Ramipril vs. Metoprolol vs. Amlodipine	2002	1,094	45.6 ± 13	0.61 ± 1.05	Hypertensive kidney disease	4.1 years	50% GFR reduction, ESKD, or death	R v M= Risk reduction 22% (1–38%, *p =* 0.04)
REIN (24)	Ramipril vs. placebo	1997	352	40.2 ± 19	5.6 ± 2.8	Non-diabetic CKD	31 months	Change in GFR, Time to ESKD, Time to overt proteinuria	52% decreased risk of overt proteinuria (*p =* 0.005). 56% decreased risk of ESKD (*p =* 0.01). No difference in rate of GFR decline
IRMA-2 (25)	Irbesartan vs. placebo	2001	590	108 ± 2	53.4 ± 2.2	Type 2 DM and Hypertension	2 years	Onset of overt albuminuria	Irbesartan 300 mg HR = 0.30 (0.14-0.61)
DETAIL (26)	Telmisartan vs. Enalapril	2004	250	91.4 ± 21.5	UAE 46.2 (4–1,011) μg/min	DM nephropathy	5 years	GFR change	
ALLHAT (27)	Lisinopril vs. Amlodipine vs. Chlorthalidone	2012	5,545	50.2 ± 8.6	n/a	Hypertensive kidney disease	8.8 years	ESKD	HR = 0.91 [0.73–1.14] vs. Chlorthalidone
**Aldosterone antagonists**									
Rossing et al. (28)	Spironolactone vs. placebo	2005	21	74 ± 6	UAE 1,566 (655–4,208) mg/day	DM nephropathy	16 weeks	Albuminuria Change in GFR	−33% reduction (−41 to −25), *p* < 0.001 −3 (−6 to 0.3) ml/min, *p =* 0.08
FIDELIO (29)	Finerenone vs. placebo	2020	5,734	44.3 ± 12.6	ACR 852 (446–1,634) mg/g	DM nephropathy	2.6 years	40% decline in eGFR, ESKD, or death	HR = 0.82 [0.73–0.93]
**Endothelin antagonists**									
ASCEND (30)	Avosentan vs. placebo	2010	1392	33.5 ± 11	ACR 163 (83-280) mg/g	DM nephropathy	4 months^*^	SCr doubling, ESKD or Death	NS
SONAR (31)	Atrasentan vs. placebo	2019	2648	44 ± 13.7	797 (462–1480) mg/g	DM nephropathy	2.2 years	SCr doubling or ESKD	HR=0.65 [0.49-0.88]
**Bicarbonate**									
de Brito-Ashurst et al. (32)	Oral sodium bicarb vs. SOC	2009	134	20.1 ± 6.5	1.7 ± 0.8	CKD stage 4	2 years	Rate of CrCl decline Rapid CrCl decline (>3 ml/min/yr ESKD	5.93 ml/min/yr vs. 1.88 ml/min/yr, *p* < 0.001 RR: 0.15 [0.06–0.40] RR: 0.13 [0.04–0.40]
UBI (33)	Oral sodium bicarb vs. SOC	2019	740	30 ± 12	0.2 (0.07–0.4)	CKD Stage 3–5	36 months	SCr doubling ESKD Death	0.36 [0.22–0.58] 0.50 [0.31–0.81] 0.43 [0.22–0.87]

*DM, Diabetes mellitus; ACR, Urine Albumin-to-creatinine ratiol; UAE, Urine albumin excretion; SCr, Serum Creatinine; CrCl, Creatinine clearance; GFR, Glomerular filtration rate; ESKD, End-stage kidney disease; CVD, Cardiovascular disease; ACEi, Angiotensin-converting enzyme inhibitor; ARB, Angiotensin receptor blocker; SOC, Standard of care; HR, Hazard ratio (95% confidence interval); RR, Relative risk (95% confidence interval)*.

* *Terminated early due to increased adverse events*.

## Aldosterone Antagonist Therapy

Spironolactone and the more selective aldosterone antagonist eplerenone have substantial antihypertensive, cardioprotective, and antiproteinuric effects even at low doses, and in the presence of combined ACEi and ARB therapy. Unlike Ang II, aldosterone is not involved in intraglomerular changes, and its mechanism of benefit may involve blockade of aldosterone effects on impaired tubuloglomerular feedback, endothelium damage and on fibrosis (34). There have been no long-term clinical trials that have studied to date the use of spironolactone or eplerenone in high-risk CKD patients, mainly due to the high risk of hyperkalemia.

Finerenone is a more selective non-steroidal mineralocorticoid receptor antagonist that reduced albuminuria in several short-term clinical trials. Preliminary studies showed that lower doses of finerenone were needed to achieve similar cardiovascular and renal effects compared to steroidal mineralocorticoid receptor antagonists (spironolactone and eplerenone), and induced less hyperkalemia. Its tissue distribution is well-balanced between cardiac and renal tissues, contrary to spironolactone and eplerenone (6 and 3 times higher concentrations in renal tissues compared to cardiac tissues, respectively) (35). This could explain the differences in mechanism and incidence of adverse events such as hyperkalemia. Recently, a large randomized clinical trial (FIDELIO) that included 5,734 patients with type 2 diabetes and CKD with albuminuria between 300 and 5,000 mg/g showed that patients with finerenone had a decreased risk of kidney disease progression or death compared to placebo, after a median follow-up of 2.6 years. Although the incidence of hyperkalemia was higher in the finerenone group, the rate of discontinuation due to hyperkalemia was relatively low (2.3%) (29).

Hyperkalemia is the main limiting factor for the use of this therapeutic group and ARB blockade. This effect could probably be mitigated with the use of the novel anti-hyperkalemia agents patiromer and sodium zirconium cyclosilicate, that have demonstrated a good tolerability and safety profile that remain consistent in the long term, in contrast with previous antihyperkalemic agents including resins that were generally poorly tolerated (36, 37) (Table 1).

## SGLT2 Inhibitors

SGLT2 inhibitors have shown remarkable additional benefits in delaying CKD progression on top of the standard RAS blockade. SGTL2i were originally developed to lower plasma glucose in type 2 diabetic patients, but large randomized controlled trials have demonstrated both renal and cardiovascular protection in both proteinuric diabetic and non-diabetic CKD patients and this effect cannot directly be explained by improved glucose control. Table 2 summarizes the clinical trials that have studied renal outcomes with SGLT2 inhibitors.

**Table 2:** Summary of kidney outcomes in clinical studies with SGLT2 inhibitors.

**Clinical Trial**	**CANVAS**	**DECLARE-TIMI**	**EMPA-REG**	**CREDENCE**	**CVD-REAL 3**	**DAPA-CKD**
SGLT2i	Canagliflozin	Dapagliflozin	Empagliflozin	Canagliflozin	Different kinds of gliflozins^*^	Dapagliflozin
*n*	10,142	17,160	7,020	4,401	35,561	4,304
GFR (ml/min/1.73 m^2^)						
Mean Range	76 >30	85 >60	74 20–90	56 30–90	91 >60	43 25–75
ACR (mg/g)						
<30 30–300 >300	70% 22% 8%	69% 24% 7%	60% 29% 11%	1% 11% 88%	N/A	200-1000: 51.3% >1,000: 48.7%
Patients	Type 2 Diabetes	Type 2 Diabetes	Type 2 Diabetes	Diabetic Nephropathy	Type 2 Diabetes	Proteinuric nephropathy (diabetic and non-diabetic nephropathy)
Renal outcomes HR [95% CI]	Albuminuria progression 0.73 [0.67–0.79]	≥40% GFR decrease, ESKD, or RR- death 0.53 [0.43–0.66]	Scr doubling, GFR <45 ml/min, dialysis, RR- death 0.54 [0.40–0.75]	Scr doubling, ESKD, or RR- death 0.66 [0.53–0.81]	>50% GFR decrease or ESKD 0.49 [0.35–0.67]	>50% GFR decrease, ESKD, RR- death 0.56 [0.45–0.68]
	≥40% GFR decrease, ESKD, or RR-death 0.60 [0.47–0.77]	ESKD 0.31 [0.13–0.79]				

* *Several kinds of glifozins (Dapagliflozin 58%; Empagliflozin 34%; Canagliflozin 6%; Pragliflozin; Tofogliflozin 2%; Luseogliflozin)*.

*Scr, serum creatinine; GFR, glomerular filtration rate; ESKD, end-stage kidney disease; RR-death, renal related death*.

The SGLT2i Cardiovascular outcome trials primarily demonstrated the efficacy of this treatment class in reducing CV risk among those with type 2 diabetes. However, secondary and exploratory analyses of these data highlighted a potential role for SGLT2i therapies in reducing adverse renal outcomes, even though the study populations were not generally considered to be at significant risk of progressive DKD (38).

The EMPA-REG OUTCOME and the CANVAS trials showed a reduced risk of new onset diabetic nephropathy and a large regression of albuminuria (return to ACR ≤ 3 mg/mmol), respectively (39, 40). Later, a large, international real-world study of patients with type 2 diabetes (CVD-REAL 3) demonstrated that initiation of SGLT2i therapy was associated with a slower rate of kidney function decline and reduced risk of major kidney events compared with other glucose-lowering drugs, with a mean follow-up was 14.9 months (41).

The CREDENCE trial was a double-blind, randomized study to assess renal treatment outcomes with canagliflozin (100 mg) among adults with type 2 diabetes and CKD. The relative risk of the composite of ESKD, doubling of the serum creatinine level or renal death was 34% lower in the canagliflozin group vs. the placebo. The components of ESKD were reduced by 32% and the risk of dialysis or kidney transplantation was reduced by 26% (42).

DAPA-CKD trial was halted early because of overwhelming efficacy, demonstrating that in patients with proteinuric CKD with or without diabetes, dapagliflozin significantly reduced the risk of 50% decline in GFR, ESKD, or death by 39% compared to placebo. The effects of dapagliflozin were similar in participants with type 2 diabetes and in those without type 2 diabetes (43).

Conclusive evidence concerning the mechanisms responsible for the renoprotective effects observed with SGLT2i is lacking, but several hypotheses have been proposed to explain the apparent slowing of DKD progression over time associated with these drugs. One of the proposed mechanisms is an improved glomerular hemodynamics due to constriction of the afferent arteriole induced by elevated adenosine levels secondary to increased membrane Na^+^/K^+^ ATPase activity, thus lowering glomerular hyperfiltration. Another mechanism is reduced tubular workload by reducing SGLT2 cotransporter activity and therefore reducing both the energy and aerobic requirements of the system. An additional mechanistic proposal is that SGLT2i agents reduce inflammation and hypoxic injury in the kidney over time. Prolonged albuminuria and high intracellular glucose levels within proximal tubular cells trigger the expression of inflammatory cytokines, growth factors, and fibrotic mediators as well as the production of reactive oxygen species. Therefore, it is possible that the renoprotective effect of SGLTi may be partly due to a reduced local inflammatory response and fibrosis.

## Endothelin Antagonists

Endothelins (ETs) are a family of vasoconstricting peptides. ET-1, the main isoform in human kidneys, is an important regulator of kidney function in health and disease, and its abnormal activation promotes kidney disease progression. It has been found to be increased in CKD patients and correlates well with kidney function and albuminuria. Two receptor subtypes are activated by ET-1; receptors type A and B. ET-A receptor provokes podocyte and mesangial lesions, oxidative stress, and inflammation leading to proteinuria and glomerulosclerosis. ET-B receptor inhibition induces fluid overload and precipitates congestive heart failure (44, 45).

Several studies in diabetic nephropathy, hypertensive kidney disease, and focal segmental glomerulosclerosis demonstrated that ET-1 inhibition can reduce proteinuria and improve kidney function (46). In a randomized clinical trial that tested avosentan, an ET-1A inhibitor, against placebo was ended prematurely after 4 months due to an increase in cardiovascular events in the avosentan arm, mainly due to fluid overload and congestive heart failure (30).

More recently, the SONAR study that included over 2,600 patients with type 2 diabetes, CKD with GFR between 25 and 75 ml/min and albuminuria between 300 and 5,000 mg/g and receiving optimum doses of RAS blockade, were randomized into receiving 0.75 mg atrasentan orally (another ET-1 receptor inhibitor that is more selective for ET-A receptor) or placebo after an enrichment period in which albuminuria decreased by more than 30% with no fluid overload. After median follow up of 2.2 years, Atrasentan reduced the risk of doubling serum creatinine or reaching end-stage kidney disease, with no significant differences with placebo in hospitalizations due to heart failure (3.5 vs. 2.6%) however there was a significant increase in fluid overload. Moreover, fluid retention was still more frequently observed in those treated with atrasentan (31).

Sparsentan, a dual endothelin-angiotensin II antagonist, showed promising results in reducing proteinuria in patients with FSGS after 8 weeks, but 16.4% of patients with sparsentan suffered from orthostatic hypotension and 12.3% had fluid retention, although none were considered serious and no patients were withdrawn from the study (47). The DUPLEX study is ongoing, which will evaluate the safety and long-term nephroprotective effects of sparsentan in FSGS patients (48) (Table 1).

## Bicarbonate

Recent data support that a component of CKD progression is mediated by mechanisms used by the kidney to increase acidification in response to an acid challenge to systemic acid-base status. An acid challenge to systemic acid-base status increases nephron acidification through increased production of endothelin, aldosterone, and angiotensin II, each of which can contribute to kidney inflammation and fibrosis that characterizes CKD (49). Several small clinical trials had highlighted a potential benefit of oral bicarbonate in delaying CKD progression. A recent multicenter, randomized, controlled trial that included 740 patients with stage 3–5 CKD examined the effect of treating metabolic acidosis with oral sodium bicarbonate compared to standard of care to delay CKD progression. Patients that received sodium bicarbonate had a 64% reduced risk of creatinine doubling, a 50% lower risk of dialysis initiation, and showed a reduction of 57% in all-cause mortality, while there was no significant risk of blood pressure elevation, heart failure, or hospitalizations (33). These results confirmed those of smaller previous studies, and show that correction of metabolic acidosis with sodium bicarbonate is a beneficial tool to delay CKD progression and improve patient survival (Table 1).

## Fibrosis, Inflammation, Other Possible Therapeutic Targets

Thus far, multiple clinical trials have been designed with the aim of delaying the progression of CKD. Most of these clinical trials have aimed to limit glomerular hyperfiltration, but there are other factors such as parenchymal cell loss, chronic inflammation, and fibrosis that are known to contribute to CKD progression and need to be addressed. Therefore, only a more holistic approach targeting all of these factors will likely achieve a more complete response and better kidney outcomes, aiming not only to delay kidney progression but also to reverse CKD.

However, despite promising results in preclinical studies, therapeutic interventions targeting “other mechanisms” in humans such as cytokines, transcription factors, developmental and signaling pathways, and epigenetic modulators, particularly microRNAs, have been disappointing, and no additional treatments are available to date.

Bardoxolone, a drug that activates nrf2, a transcription factor that controls various cytoprotective proteins, was studied to target inflammation and oxidative stress. Bardoxolone improved GFR in type 2 diabetics with CKD, an effect that persisted throughout the 52 weeks of study (50). Nevertheless, a follow-up phase III study of bardoxolone that targeted renal events was stopped for safety concerns because of excessive serious adverse events and mortality in the bardoxolone arm (51). No further studies targeting this pathway are currently underway.

Novel therapies that are currently under evaluation target renal fibrosis, including the anti-fibrotic agent pirfenidone. Although its mechanism of action is not fully understood, it interrupts the TGF-β pathway. The main clinical trial was done in patients with diabetic nephropathy, with significant improvement in GFR compared to placebo, however it failed to reduce proteinuria (52). Another study conducted in FSGS patients also observed a failure to reduce proteinuria with pirfenidone (53), suggesting that this drug can ameliorate progression of kidney disease but without reducing proteinuria, due to either a structural improvement in interstitial injury or due to a hemodynamic effect on GFR.

Pentoxifylline is being studied for its inflammatory modulation and anti-oxidative stress, and has shown in several studies a significant reduction in proteinuria when it was added to RAS blockade in CKD patients (54).

## Biomarkers

It is still challenging to predict progression in CKD. This is mainly due to a scarcity in sensitive and specific biomarkers for predicting CKD progression early. The development of end-stage kidney disease may take several years, therefore surrogate endpoints such as albuminuria and serum creatinine have been increasingly used in trials over hard endpoints to predict CKD progression (55–57). Although albuminuria may allow for an early management of CKD, the reduction of albuminuria does not always translate into slowing CKD progression, and also not all causes of CKD develop albuminuria (58). Several studies have shown that using GFR decline of 30 or 40% as alternative surrogate endpoints of CKD progression may allow a more prompt detection, allowing for trials with shorter follow-up periods (59, 60). Nevertheless both biomarkers are established kidney injury markers and we are still in search for more biomarkers that identify patients at high risk of progression at an earlier phase, allowing for earlier therapeutic intervention, as well as more specific biomarkers that could provide better tools for individual adjustment of treatments.

Dickkopf-3 (DKK3) is a stress-induced, renal tubular epithelial-derived glycoprotein that induces tubulointerstitial fibrosis through its action on the canonical Wnt/β-catenin signaling pathway. Elevated urinary DKK3 levels identify patients at high risk of rapid decline in GFR during the following 12 months regardless of the etiology of CKD. Moreover, uDKK3 levels are directly associated to the degree of tubulointerstitial fibrosis in kidney biopsies (61). In recent studies, it has been observed that high pre-surgery uDKK3 levels were an independent predictive factor for the development of postoperative AKI and for the subsequent loss of kidney function (62).

Moreover, several biomarkers of cardiovascular risk in CKD have been assessed, and could be classified into prognostic biomarkers, such as cardiac troponins and NT-ProBNP, Cystatin C, β2-Microglobulin, Galectin-3 and markers of inflammation or tissue remodeling such as matrix metalloproteinases (MMPs), and predictive biomarkers that predict response to treatments, such as proteinuria, insertion/deletion polymorphisms of the ACE gene, MMP levels, and renal resistive index in kidney ultrasound (63).

Some biomarkers such as kidney injury molecule (KIM-1) (64), neutrophil gelatinase-associated protein (NGAL) (65), apolipoprotein A-IV (apoA-IV) (66), soluble TNFα receptor 1 (TNFR1) (67), and soluble urokinase receptor (suPAR) (68) have been evaluated as potential biomarkers of kidney disease progression. Unfortunately, none of them have demonstrated to add an additional benefit to serum creatinine and albuminuria. These tubular biomarkers were described in the acute ischemia-reperfusion damage models and do not reflect tubulointerstitial fibrosis. There is a constantly increasing number of studies that are revealing novel biomarkers and potential therapeutic targets by using micro-RNA analysis, proteomics, peptidomics, and urinary transcriptomics.

## Future Lines of Treatment: Renal Regeneration

There is strong evidence from animal studies that suggests that interstitial fibrosis could be reversed and therefore could be a therapeutic target in the prevention of CKD progression (69–71). The concept of kidney regeneration is emerging, and it could be achieved by using growth factors, or multipotent cells could be directed to regenerate kidneys with chronic lesions.

Stem cell-based regenerative therapy is an alternative future treatment modality. There have been studies performed with hematopoietic stem cells, mesenchymal stem cells, and endothelial progenitor cells. Patients with CKD have a decreased capacity for kidney regeneration with an altered function of endothelial progenitor cells, and several studies in animal models with CKD suggest a regenerative beneficial effect of these cell-based therapies (72, 73).

A meta-analysis of several experimental models found that stem cell-based therapy prevented progression of CKD with decreased proteinuria (74). Mesenchymal stem cells are being used in kidney transplant patients to increase immunosuppression and improve regeneration (75). On the other hand, studies involving hematopoietic stem cell-based regenerative therapies are mainly in lupus nephritis and are uncontrolled (76).

## Conclusions

Progression of CKD to ESKD carries a high burden on cardiovascular morbidity and mortality. We need to improve our understanding of early mechanisms of CKD which will consequently enable early therapeutic intervention and delay CKD progression or even reverse it. In addition to RAS blockade, SGLT2 inhibitors and bicarbonate therapy have proved to retard CKD progression, and new drugs targeting fibrosis and inflammation, as well as regenerative therapy may enhance these effects in our goal to mitigate CKD progression and consequently cardiovascular and global mortality.

## Author Contributions

AS, CC-C, and GF-J have designed, written, and reviewed this paper. All authors contributed to the article and approved the submitted version.

## Conflict of Interest

The authors declare that the research was conducted in the absence of any commercial or financial relationships that could be construed as a potential conflict of interest.

## Supplementary Material

Supplementary Figure 1 Contributors to progression of Chronic Kidney Disease.
