# Supplementary figures and images for: Therapeutic Insights in Chronic Kidney Disease Progression
Source: Front Med (Lausanne). 2021 Feb 23;8:645187. doi: 10.3389/fmed.2021.645187 (PMC7940523; doi:10.3389/fmed.2021.645187)

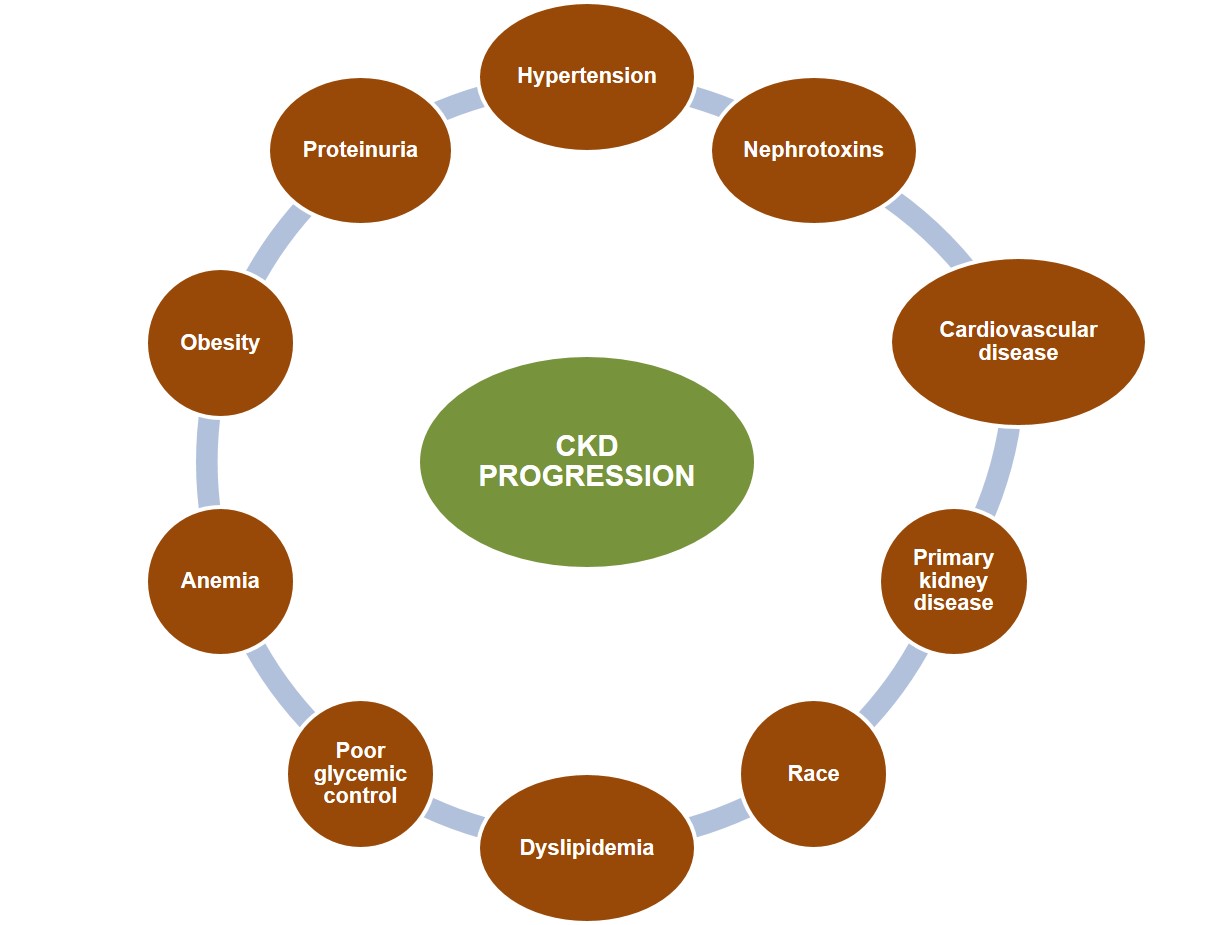

Supplement: Supplementary Figure 1 — Contributors to progression of Chronic Kidney Disease. [file Image_1.JPEG]
